# Supplementary material for: A survey of mapping algorithms in the long-reads era
Source: Genome Biol. 2023 Jun 1;24:133. doi: 10.1186/s13059-023-02972-3 (PMC10236595; doi:10.1186/s13059-023-02972-3)
Supplement: Supplementary file 1 — Additional file 1. Additional figures and details on tools’ strategies. [file 13059_2023_2972_MOESM1_ESM.pdf]

Additional File 1

Details on subsampling techniques In Supplementary Figure S1, we present an example of the difference in the selected bases between a minimizer approach and a syncmer approach.

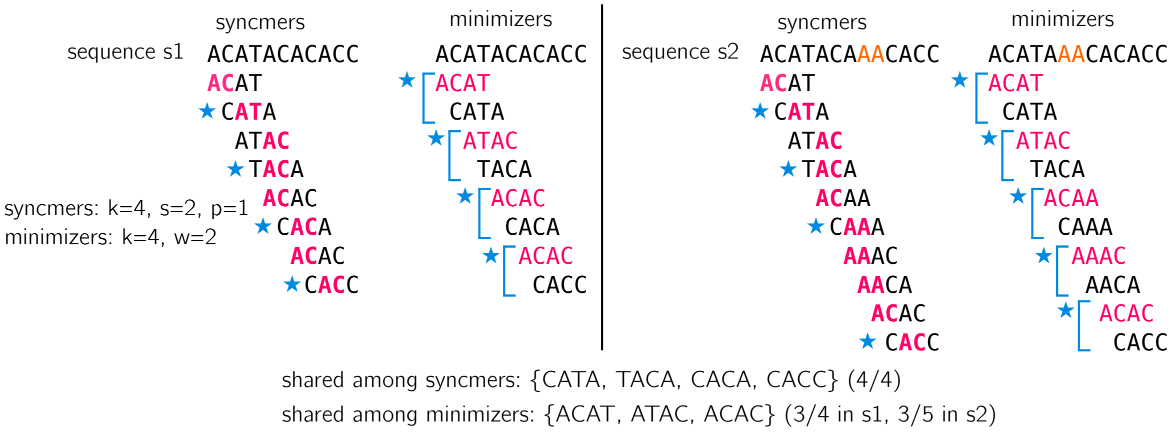

Figure S1: Usage syncmers and minimizers for comparing two similar sequences. Sequences s1 and s2 differ by a AA insertion in orange in S2. We show how selected syncmers and minimizers do not produce the same sets of representative k-mers and therefore yield different fractions of shared k-mers between s1 and s2. The k-mer size is 4, the s-mers in syncmers (smallest showed in pink, we choose the lexicographic order) are of size 2, and in this example we require that the smallest s-mer appears at the first position of the k-mer. Minimizers have windows of size 2, materialized in blue, with the minimizer in pink. The selected k-mers are highlighted using a blue star.

Graphmap's indexing strategy for fuzzy seeds Graphmap builds two hash indexes from two types of shapes , called `6-1-6` and `4-1-4-1-4`. As shown in Supplementary Figure S2, for each position is seeded (no subsampling). A seeded key corresponds to the subsequence at a given position of the reference when applying the shape mask: each *don't care* (*) base is skipped.

Then, for each read in the query, several lookup keys (for mismatch, deletion and insertion) are built from a shape (Supplementary Figure S3). To that extent, the lookup key treats the *don't care* base in three different ways. The mis(match) shape has the same behaviour as the indexed key, i.e., the *don't care* base are skipped. The insertion shape skips two bases: the initial *don't care* base and the base next to it. Finally the deletion shape will simply build the key and keep all the base including the *don't care* base. In total, for a number d of *don't care* base, 3^d different keys are built per shape.

Graphmap's backbone graph for LCSk Because of the possible spurious matches that occur because of the ambiguous bases, `Graphmap`'s fuzzy seeds require more treatments to find proper chains. A first step after seeding finds groups of anchors representing longer shared subsequences between the query and the reference, on which is applied LCSk.

Anchors are placed in a vertex-centric positional graph of k-mers, in which k-mers in both sequences appear, and share an arc if they are directly consecutive (or consecutive up to a distance parameter)[1]. Most weighted paths of anchors (i.e. supported by the query and the reference) are found in this graph and output as shared subsequences. After the LCSk pass, a L1 linear regression step is applied to fit a straight line with a 45 degree slope and remove outliers, especially in the beginning and end of the chain (see case 4 in Figure 5 in the main text). Note that other methods use graphs built over anchors as backbones to the chaining and alignment steps without fuzzy seeds [2, 1].

Minimap2's complete formula for regular and large gaps size

[1]NB: this is different from a de Bruijn graph since nodes with similar contents can be repeated

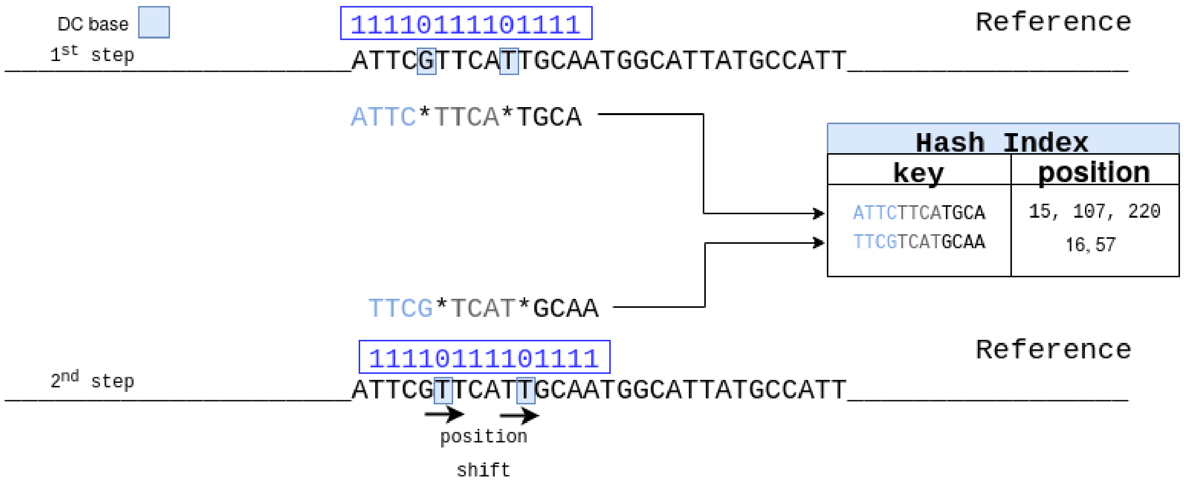

Figure S2: Indexing scheme for fuzzy seeds allowing indels and substitutions in Graphmap. In the figure the shape 4.1.4.1.4 is represented. The zeros represent the don't care positions of the shape. The shape is then applied for each position of the genome. The substring built from the shape is used as key inside a hash index. Each key will correspond to one or more positions on the reference.

- For regular gaps size:

$$f(i) \quad = \quad \max\{ \max_{\substack{i>j\geq 1 \\ x_i-G<x_j\leq x_i \\ y_i-G<y_j<y_i}} f(j) + \min\{d_{j\,i}, w\} - \gamma_r(g_{j\,i}), w\}$$

  The property $x_i - G \leq x_j \leq x_i$ and $y_i - G \leq y_j < y_i$ is equivalent to $y_j < y_j$, $x_j \leq x_i$ and $e_{j\,i} < G$.

- For large gaps size:

$$f'(i) \quad = \quad \max_{\substack{i>j\geq 1 \\ x_i-G\leq x_j\leq x_i-w \\ y_i-G\leq y_j\leq y_i-w}} f'(j) + w - \gamma_l(g_{j\,i}, d_{j\,i})$$

where

$d_{j\,i} = \min\{y_i - y_j, x_i - x_j\}$		Smaller "distance" between the two anchors. This is not really a distance by definition : for $(x_i, y_i) = (-n, 0)$, $(x_j, y_j) = (0, 0)$ and $(x_k, y_k) = (0, n)$, we have $d_{i\,j} + d_{j\,k} = 0 < n = d_{i\,j}$.
$e_{j\,i} = \max\{y_i - y_j, x_i - x_j\}$		Discrete Chebyshev distance between the two anchors
$g_{j\,i} = \lvert(y_i - y_j) - (x_i - x_j)\rvert$		Gap length (or Manhattan distance between the diagonals passing by the two anchors)
$\gamma_r(g) = 0.01 \times w \times g + 0.5 \log_2 g$		
$\gamma_l(g, d) = c_1 \times g + c_2 \times d + \log_2 g$		where c_1 and c_2 are parameters

Hough transform principle Applying the Hough transform means going from the $S1 = (query, reference)$ space to the Hough S_2 space of coordinates. If a line $(y = ax + b)$ exists in S_1, it is a point of coordinates (a, b) in S_2 (practically, polar coordinates are used for technical reasons). All possible lines intersecting a point in S_1 can be translated in S_2 as a sine wave. Multiple anchors give multiple points in S_2, and the intersection of possible sinusoids intersecting the different points in S_2 correspond to a line roughly intersecting the initial anchors in S_1. The Hough space is rasterized, and by counting

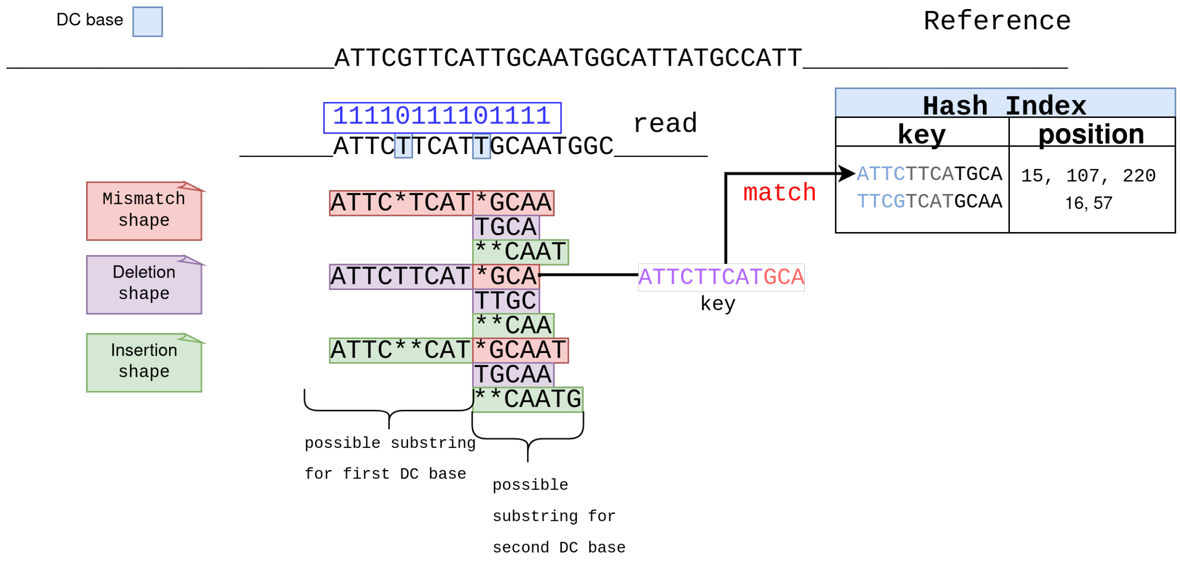

Figure S3: Query in Graphmap, different possible sequences can be matched using a single key. As we can see, there are three types of look-up shapes, and each of them is used to reconstruct a different substring. Each type corresponds to three phenomena that can occur with errors in sequencing, namely substitution, substitution + 1 insertion, and substitution + 1 deletion. Here, two don't care bases are present and nine substrings can be obtained. In this example the substring obtained from the substitution + insertion shape and the mismatch leads to a match with the reference.

and weighing the possible solutions in S_2, lines can be deduced in S_1. Contrary to linear regression which would output the best line explained by the seed distribution in S_1, here an arbitrary number of straight lines can be output and considered (see Supplementary Figure S4 for an overview of the steps).

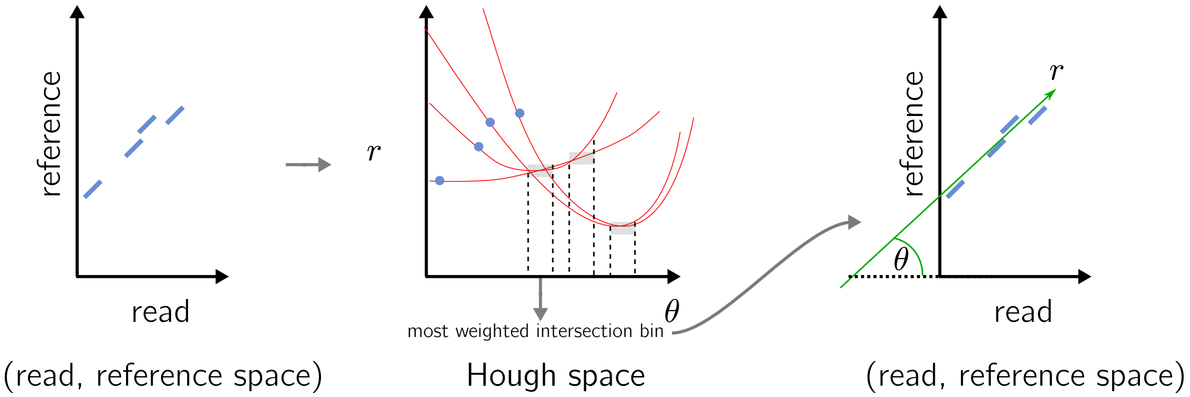

Figure S4: An overview of the Hough transform steps.

References

[1] Bo Liu, Dengfeng Guan, Mingxiang Teng, and Yadong Wang. rHAT: fast alignment of noisy long reads with regional hashing. *Bioinformatics*, 32(11):1625–1631, 11 2015. arXiv:https://academic.oup.com/bioinformatics/article-pdf/32/11/1625/22645531/btv662.pdf, doi:10.1093/bioinformatics/btv662.

[2] Ze-Gang Wei, Xing-Guo Fan, Hao Zhang, Xiao-Dan Zhang, Fei Liu, Yu Qian, and Shao-Wu Zhang. kngmap: sensitive and fast mapping algorithm for noisy long reads based on the k-mer neighborhood graph. *Frontiers in Genetics*, page 988, 2022.
